# Hock and Moselle

**Published:** 1907-06-29

**Authors:** Robert Gray

**Affiliations:** Past Chairman, Wine and Spirit Association. Founder and Trustee, Wine and Spirit Trade's Benevolent Society


HOCK AND MOSELLE.

*Sir*,—I thank you for your article and report on wine, which is a valuable contribution to the current literature on the subject, and should be in the hands of every wine-merchant.

I, as a layman, accept your medical conclusions, because they are the dispassionate statements of medical men, and bear upon them the impress of truth, but why, oh, why, did you not have the report edited by an *independent* expert connected with the trade. I presume you have had the collaboration of someone with technical knowledge of wine, but why did he let you make, on page 288, a mistake in the name of one of the Médoc growths, Haut, not Haute Lafite, and in the spelling of Sharzberger, a German wine also, on page 292, a double mistake in a German wine, and why did you let him mislead you with regard to the supply of genuine French and German wine. I refer more particularly to the remarks on pages 289 and 295. I hold no brief for German wine and would as soon sell one kind as another, but I question if you would find even *one* unprejudiced expert who would not tell you that you have done less than justice to German wines. I am, dear Sir,

Yours truly,

Robert Gray.

Past Chairman, Wine and Spirit Association.
Founder and Trustee, Wine and Spirit Trade's Benevolent Society.

.˙. Mr. Gray calls our attention to two typographical errors on page 288. We may state that both of these were as a mattter of fact observed in proof and were corrected, but by an oversight the correction was not made in the final imprint. The first was in the case of the Ch. Haut-Lafite, which was printed Ch. Haute Lafite. We may add that the full name of this wine is Ch. Smith Haut-Lafite. While thanking our correspondent we would point out that he himself has made a much more serious mistake, involving wrong classification. Mr. Gray refers to this wine as a Médoc growth. It is as a matter of fact not a Médoc wine, but a Red Graves. We must presume that it is to this wine that Mr. Gray referred, as all the other names are perfectly correct. The second typographical error was where a Moselle was erroneously spelt "Schwarzberger" instead of "Scharzberger." The same applies to the spelling of the name of the same wine on page 292. With regard to Mr. Gray's remarks concerning the supply of genuine French and German wines we regret that we must adhere to the view taken by us. It is a matter of common knowledge that the quantity of wine produced in Germany is exceedingly small compared with that in France, and the law of supply and demand therefore is no doubt responsible for the fact that the amounts of Hock and Moselle of certain brands sold is largely in excess of the amounts produced in the very limited areas in which they are grown. We could mention names of brands of Moselle, for instance, of which more is consumed in half-a-dozen London clubs than is produced in the vineyards from which they are supposed to originate.—Ed. The Hospital.

